# Proliferation of enterococci in sargassum can contribute towards sand and water quality impairment

**DOI:** 10.1007/s10661-026-15872-3

**Published:** 2026-09-02

**Authors:** Afeefa A. Abdool-Ghany, Kevin Huang, Matthew Roca, Eric Saba, Jiayu Li, Helena M. Solo-Gabriele

**Affiliations:** 1 Department of Chemical, Environmental and Materials Engineering, University of Miami, 1251 Memorial Drive, Room 508, Coral Gables, FL 33146 USA; 2 Department of Biological Sciences, Florida International University, Miami, FL USA; 3 Department of Mechanical and Aerospace Engineering, University of Miami, Coral Gables, FL USA

**Keywords:** Beach, Rainfall, Leachates, Mesocosm, Seaweed, Wrack

## Abstract

The fecal indicator bacteria (FIB), enterococci, are used to evaluate the safety of beach waters and beach sands. The increasing quantities of the holopelagic macroalgae *Sargassum fluitans* and *Sargassum natans* (referring to herein as “sargassum”) along beaches that face the tropical Atlantic Ocean have motivated field observational studies suggesting that the levels of enterococci at beaches are influenced by the presence of stranded sargassum at beaches. The objectives of this study were to evaluate whether enterococci multiply in sargassum and to evaluate the impact of sargassum on enterococci levels within underlying sand and rainwater leachates. Results show considerable regrowth of enterococci in sargassum under natural rainfall and sunlight conditions. Levels of enterococci in sargassum were initially at 2.3log_10_ and increased to 5.3log_10_ (original units of colony-forming units per gram). By the end of the 70-day study, after an 18-day period of no rainfall, the elevated levels persisted in sargassum at 4.8log_10_. Enterococci levels in sand and rainwater leachates impacted by sargassum were significantly elevated in comparison to the control which did not have a sargassum layer (*p* < 0.01). Overall results show that enterococci multiplication was associated with the presence of sargassum*,* contributing towards impaired sand and water quality. This information is of significance for beach managers when they address the need for sargassum removal when strandings are excessive.

**Supplementary Information:**

The online version contains supplementary material available at https://doi.org/10.1007/s10661-026-15872-3.

## Introduction

The fecal indicator bacteria (FIB), enterococci, are used to evaluate the recreational safety of marine waters. The traditional paradigm driving FIB measurements is that of a waterborne source of wastewater contamination, such as through an ocean outfall or river discharges contaminated with sewage. If FIB is measured at a beach water site above a set threshold, then the site is presumed to be contaminated with sewage and/or fecal waste, and advisories are issued to discourage swimming. Guidelines for thresholds are provided at the federal level (US EPA 2012) and these guidelines are interpreted and adopted at the State level. For the State of Florida, USA, the guideline for Florida beaches is a geometric mean of 35 colony-forming units (CFU) per 100 mL and a single-sample maximum of 70 CFU/100 mL.

Studies have shown, however, that the beach shore can also serve as a non-point source of FIB (Vogel et al., 2017), with beach sands harboring elevated bacteria and pathogens, especially in the supratidal zone just above the high tide line (Feng et al., 2015; Shah et al., 2011; Wright et al., 2011). Sources to beach sands have included animals such as birds (Byappanahalli et al., 2015; Lu et al., 2011; Oshiro & Fujioka, 1995) and dogs (Wright et al., 2009; Martine et al., 2024; Dhar et al., 2025). In addition to direct animal inputs, recent work suggests that decaying organic material in the wrack line can also promote the persistence and regrowth of enterococci (Abdool-Ghany et al., 2022; Kalvaitienė et al., 2024; Quilliam et al., 2014) and other fecal bacteria (Badgley et al., 2011) by providing both nutrients and moisture microhabitats (Shibata et al., 2004). Due to the recognition that FIB can also impact beach sand, the World Health Organization (WHO, 2021) has recommended a guideline level for enterococci in beach sand of 60 CFU/g.

With the dramatic increases in holopelagic sargassum inundations (specifically *S. natans* and *S. fluitans*) along coasts that border the tropical Atlantic Ocean (Rodríguez-Martínez et al., 2025), studies have attributed sargassum as the cause of elevated levels of enterococci in water (Tomenchok et al., 2021) and beach sand (Abdool-Ghany et al., 2022, 2026a). For example, Abdool-Ghany et al. (2022) conducted a year-long study at Key Biscayne Beach, Florida, measuring fecal indicator bacteria levels before, during, and after the COVID-19 beach shutdown. The study found that decomposing sargassum harbored and likely promoted the regrowth of enterococci, contributing to elevated bacterial levels in surrounding sand and nearshore waters. During the shutdown, when human activity was minimal, high enterococci levels were confined to the zone where sargassum was stranded; once the beach reopened, human foot traffic was believed to disperse bacteria across the supratidal and intertidal zones, demonstrating that stranded sargassum is associated with a persistent environmental source of contamination. Most studies focused on the beach shore are based upon field monitoring and establish the attributions by association. Studies exist that have evaluated seawater and sand mesocosms (but not wrack) with applicable findings. For example, Brown and Boehm (2015) evaluated sand and seawater mesocosms without wrack and found greater persistence of enterococci in unwetted sand and under dark conditions. Only a few studies exist (that we are aware of) have included wrack under controlled laboratory conditions. These studies utilized mesocosms to evaluate impacts of wrack on FIB levels in seawater (Quilliam et al., 2014) and sediment (Imamura et al., 2011). Quilliam et al. (2014) showed that brown seaweed (*Laminaria saccharina*) enabled the persistence of *E. coli* in seawater. Imamura et al. (2011) demonstrated persistence of FIB over a period of five days with no rain (average temperature of 18 °C).

To our knowledge, no previous work has examined wrack-sand systems in long-term, controlled experiments that incorporate natural rainfall conditions commonly experienced in areas with seasonal wrack accumulation. The current study fills the information gap through mesocosm experiments that simulate the supratidal beach sand environment. The objective of the mesocosm experiments was to determine whether enterococci can grow on sargassum and to evaluate the impact of sargassum on enterococci levels within underlying sand and rainwater leachates. Specifically, this current study evaluated the hypothesis that sargassum acts as both a source and amplifier of enterococci, and that its presence creates conditions favorable for bacterial persistence. This study involved the analysis of two sets of mesocosms, one with sand only (SO) as a control, and another set with sargassum layered over sand (SS) as the experimental. Enterococci levels were measured over time in rainwater, sargassum, sand, and leachates, and compared between the SS and SO mesocosms. Results provide evidence of the ability of enterococci to grow in sargassum, offering information needed for beach management in efforts to limit enterococci levels in water and sand.

## Methods

### Sargassum collection

**Fig. 1:**
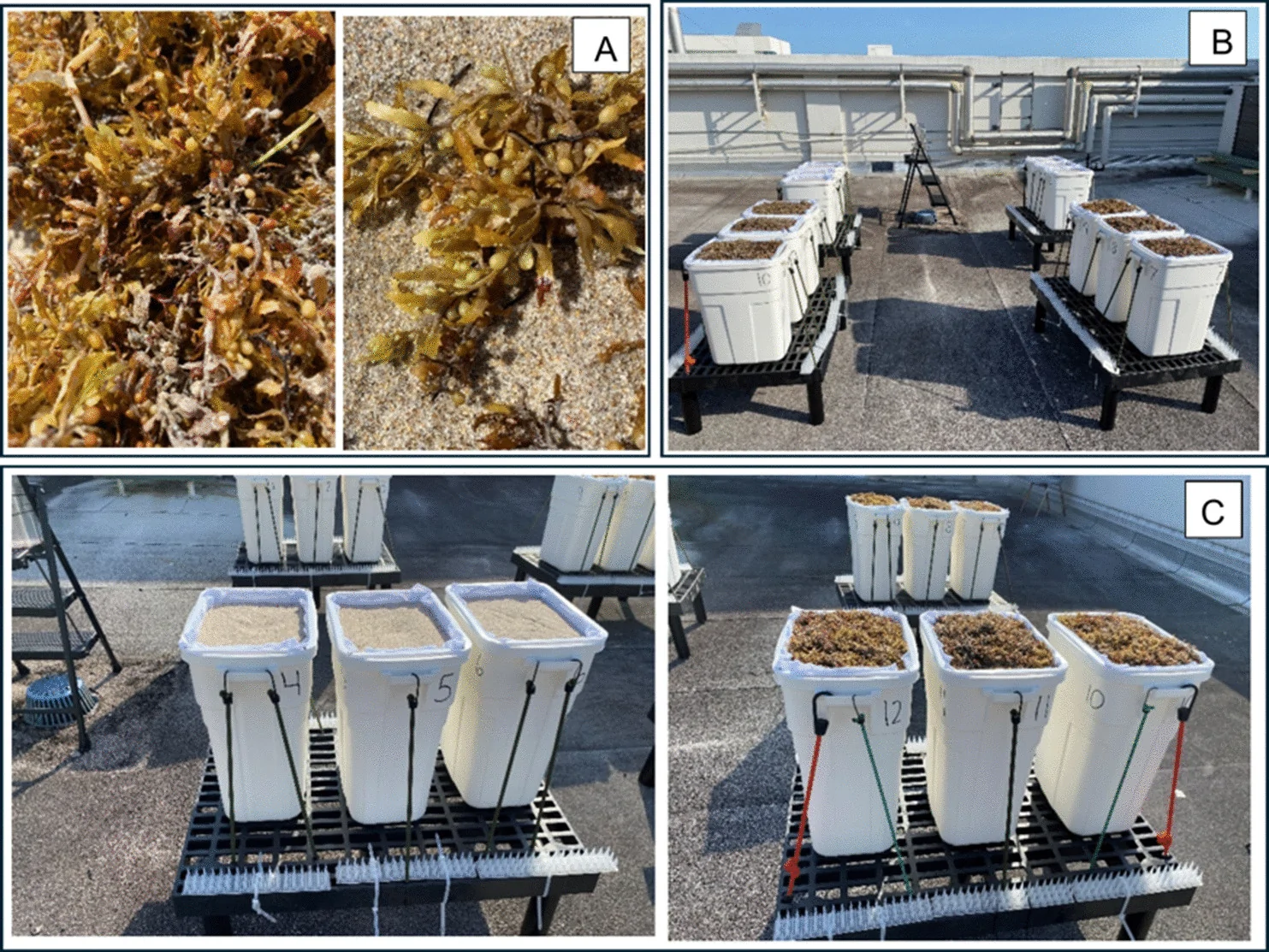
Close-up photos of stranded sargassum used in this study, illustrating predominantly *Sargassum fluitans* (**A**). Photo of entire mesocosm system illustrating the 12 mesocosms, bird deterrents, and one of the two rain gauges at a distance (**B**). Closeup photos of 3 of the 6 SS mesocosms and 3 of the 6 SO mesocosms (**C**)

Freshly stranded sargassum (confirmed as predominantly *Sargassum fluitans*) was collected on March 13, 2024, from an ocean-facing beach in South Florida (Lauderdale-by-the-Sea Beach, 26° 11′ 31.29 N, 80° 05′ 40.18W). The samples were characterized by a golden color typical of the freshly stranded macroalgae (Vázquez-Delfín et al. 2021) (Fig. 1, panel A) and a moisture content of 57% which indicates that some drying may have occurred. Samples were collected at about 11 am and were transported in plastic-lined coolers to the laboratory for mesocosm setup within 1.5 h of collection. In addition to the sargassum, sand from the supratidal zone was also collected in a separate plastic lined cooler.

### Mesocosm setup

Twelve mesocosms were set up outdoors on the flat roof of a five-story building (McArthur Engineering Building of the University of Miami following methods described in Abdool-Ghany et al., 2026b) to allow for natural rainfall and sunlight, and to avoid contamination from street-level activities (Fig. 1B). These 12 mesocosms included six controls (collectively called SO for sand-only mesocosms) and six experimental (collectively called SS for sand + sargassum mesocosms). Each of the 12 mesocosms was fitted with a plastic tray strainer (28.4 cm × 22.0 cm × 4.2 cm) that was lined with felt. Beach sand was placed on top of the felt (2 kg each) to a depth of 3 cm for both the SO and SS mesocosms. For the six SS mesocosms, an additional layer of 0.5 kg of sargassum was overlaid on the sand. Each of these plastic tray strainers was fitted tightly on top of a reservoir which captured rainfall that infiltrated through the top layers (Fig. 1B and C).

Three bird deterrent systems were utilized around the mesocosms to avoid bird fecal waste including reflective rods, fence spikes, and an ultrasonic repellant. No birds nor evidence of bird fecal material was observed throughout the course of the mesocosm experiments. In addition to bird deterrents, two sanitized standard rain gauges (monitored daily between 9 and 10 am) were placed on each side of the mesocosms to document rainfall depths and to obtain rainfall samples for enterococci measurements.

### Sample collection from mesocosms

Upon receipt of the sargassum and sand at the laboratory, each was composited and set to represent Day 0 (March 13, Wednesday). Samples were collected from the mesocosms between 9 and 10 am on the following Saturdays and Tuesdays and following each rainfall day except for May 4 to the last day of sampling on May 22 when samples were collected only after rainfall days. The mesocosms were run for a total of 70 days.

Rainfall was detected in the rain gauges during 10 days of the 70 days (total = 19.36 cm; range 0.24–9.13 cm per rain day). Of these 10 days, rainfall was sufficient to generate leachate on 9 of the 10 days. During these 9 days, the leachate volumes captured in the reservoirs were measured volumetrically for small volumes (< 100 mL per reservoir) or by weight for large volumes (> 100 mL).

All samples were collected as composites of aliquots removed from the corresponding set of mesocosms. Rainwater composites consisted of equal volumes from each rain gauge combined in a sterile container and then utilized for enterococci measurements. For the leachates, composites were prepared in a sterile container per event, one composite corresponding to the leachate for the six SO mesocosms and another composite corresponding to the leachate for the six SS mesocosms. After collection, any remaining leachate water in the reservoir was discarded to avoid carryover between events. For sand, composites consisted of collecting aliquots from the six corresponding mesocosms using a sterile stainless-steel spoon. For sargassum, aliquots were clipped from the six SS mesocosms with sanitized stainless-steel scissors. Prior to analyses, samples were homogenized by mixing in Whirl–Pak bags and then split for: (1) moisture content by gravimetric analysis (before drying and after 24 h of drying in a laboratory oven set at 100 °C) and (2) enterococci enumeration. Moisture content was defined as evaporated water mass divided by wet mass of sargassum or sediment.

### Enterococci enumeration

Enterococci were measured by membrane filtration for water samples or liquid extracts from sand and sargassum. The filters (GN-6, Pall Filters, 47 mm diameter, 0.45 µm pore size) were placed on mEI agar at 41 ± 0.5 °C for 24 h as per standardized procedures (US EPA 2014, Method 1600). Colony-forming units with characteristic morphology were counted and back-calculated to CFU per dry gram using the paired moisture content measurement or to CFU per 100 mL using the sample volumes considering dilutions.

Liquid extracts from sand and sargassum were prepared using the methods documented by Boehm et al. (2009) for sand and Abdool-Ghany et al. (2022) for sargassum. Briefly, sand extracts were produced by aseptically weighing about 16 g wet mass within a sterile bottle. One hundred milliliters of sterile phosphate buffered saline (PBS) were added to the bottle and the bottle was shaken for 2 min and the sample was allowed to settle for another 2 min. For sargassum extracts, about 4 g wet mass of sargassum was aseptically weighed into a sterile Whirlpak bag. Two hundred milliliters of PBS were added, and the sample was processed by pressing the sargassum and PBS solution between the thumb and fingers for 2 min. Like the sand, the sample was allowed to settle for another 2 min prior to further dilution, if necessary, and filtration.

Because sample concentrations varied widely over the course of the experiment, the filtered through the membranes changed over time to ensure results remained within quantifiable detection ranges. For rainfall, 100 mL volumes were preferred due to the anticipated low levels of enterococci. When sample volumes from the rain gauges were less than 100 mL the volume available was utilized for analysis (with 20 mL as the smallest volume). For leachates from both sets of mesocosms, sample elution volumes used for membrane filtration ranged from 0.05 to 100 mL. For sediment, filtered eluate volumes typically ranged from 1 to 20 mL, with volumes as small as 0.5 and 0.05 mL when enterococci concentrations were high. For sargassum, volumes generally ranged from 1 to 10 mL, with serial dilutions extending to 0.5, 0.05, 0.005, and 0.0005 mL during periods of elevated concentrations.

### Statistical analyses

All statistical analyses were conducted in R (version 4.4.3) using base functions and the stats and multcomp packages. Descriptive statistics (mean, median, standard deviation, and interquartile range) were computed for each matrix. Box plots were utilized to illustrate differences among the data sets. For the box plots, the line within the box represents the median. The box boundaries represent the upper and lower quartiles. The error bars represent 1.5 times the interquartile range with circles outside the error bars representing outliers. Temporal trends were evaluated qualitatively through time-series plots.

The normality of log₁₀-transformed enterococci data for each matrix was assessed using the Shapiro–Wilk test. The data were log_10_ transformed due to the large range of enterococci levels observed in this study. Most log_10_ transformed datasets (sand-only, sand + sargassum, leachate, and rainfall) met normality assumptions (*p* > 0.05), while the sargassum dataset showed some non-normality (*p* = 0.001). Because ANOVA is robust to deviations from normality and all other groups were normally distributed, parametric tests (one-way ANOVA and Tukey’s HSD post hoc) were selected as the primary analyses for the log_10_-transformed data. For verification, nonparametric Kruskal–Wallis tests were also performed and produced consistent significance patterns across matrices. Due to the wide range of dilutions used, all sand and leachate samples were within quantifiable limits. One of the 20 sargassum samples were below detection limits (3 days after the initiation of the experiment). The non-detect value was treated as missing and excluded from log_10_ analyses. Post-hoc comparisons using Tukey’s HSD test to identify pairwise differences between sample types (sargassum, SS sand, SO sand, SS leachate, SO leachate, and rainfall) used a significance threshold of *α* = 0.05 for all hypothesis tests. Data sets with *p*-values less than 0.05 were considered statistically different.

To assess whether moisture content (*MC*) influenced enterococci concentrations, an analysis of covariance (ANCOVA) was performed using sample type (sargassum, SS sand, and SO sand) as a categorical predictor and *MC* as a continuous covariate. Interaction terms between sample type and moisture content were evaluated to test whether the effect of moisture differed by matrix.

Pearson correlations (*R*^2^) were evaluated between enterococci levels within the different matrices (e.g., enterococci levels in sargassum versus enterococci levels in the SS sand) and between moisture content and levels of enterococci in the corresponding matrix (e.g., moisture content of the sargassum compared to the levels of enterococci in sargassum). Correlations below 0.3 were considered weak, above 0.3 to 0.7 were considered moderate, and above 0.7 were considered strong. Correlations were considered significant for *p*-values less than 0.05.

## Results

**Fig. 2:**
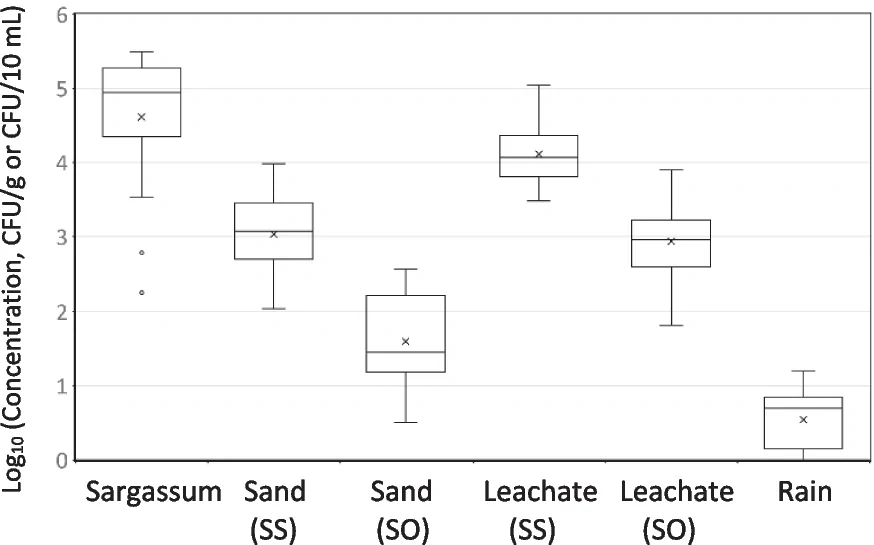
Box and whisker plot of sargassum (*n* = 20), sand (*n* = 20 for SS and *n* = 20 for SO), leachates (*n* = 8 for SS and *n* = 8 for SO), and rain (*n* = 9) concentrations in the SS and SO mesocosms. Units for sargassum and sand in CFU/g. Units for leachates and rain in CFU/100 mL. The × within the box corresponds to the mean of the log_10_ transformed enterococci concentration (equivalent to the geometric mean without transformation). The lines within the boxes correspond to the medians. The edges of the box plot correspond to the upper and lower quartiles of the range. The error bars represent the 1.5 times the interquartile range and points outside the error bars represent outliers

Results show that sargassum consistently had the highest levels of enterococci (geometric mean = 4.1 × 10^4^ CFU/g; log₁₀ = 4.61), compared to sand in the SS mesocosm (geometric mean = 1.1 × 10^3^ CFU/g; log₁₀ = 3.03, *p* < 0.001) and sand in the SO mesocosm (geometric mean = 40 CFU/g; log₁₀ = 1.60, *p* < 0.001) (Fig. 2). Among the sands, the concentrations in the SS mesocosm were significantly larger than those in the SO mesocosm compared to the SO mesocosm (mean difference = 1.44 log₁₀, *p* < 0.001). Similarly, for the leachates, the concentrations in the SS mesocosm (geometric mean = 1.3 × 10^4^ CFU/100 mL; log₁₀ = 4.11) were significantly larger than the concentrations in the SO mesocosm (geometric mean = 8.7 × 10^2^ CFU/100 mL; log₁₀ = 2.94; *p* = 0.008). Rainfall was characterized by the lowest enterococci concentrations (geometric mean = 3.5 CFU/100 mL; log₁₀ = 0.54) compared to both leachates (*p* < 0.001).

**Fig. 3:**
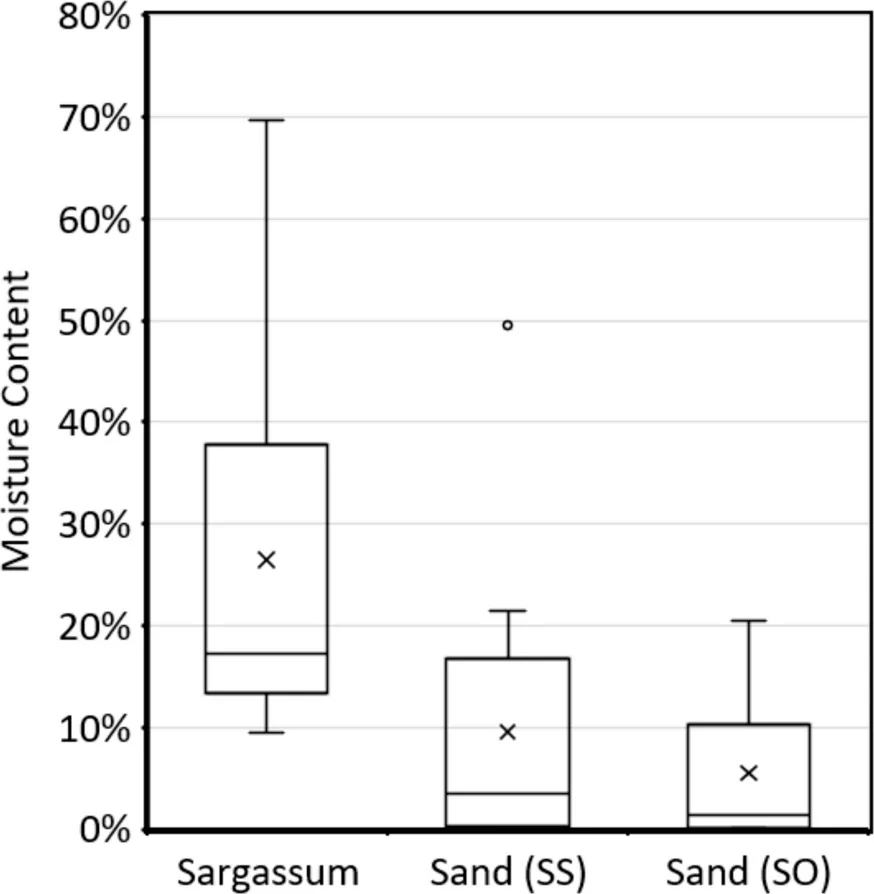
Box and whisker plot of sargassum (*n* = 20) and sand from the SS (*n* = 20) and SO (*n* = 20) mesocosms. Moisture content was analyzed for each time point that a sample was collected for enterococci analysis, as illustrated in Fig. 4. The definition of the boxes and error bars is provided in Fig. 2

**Fig. 4:**
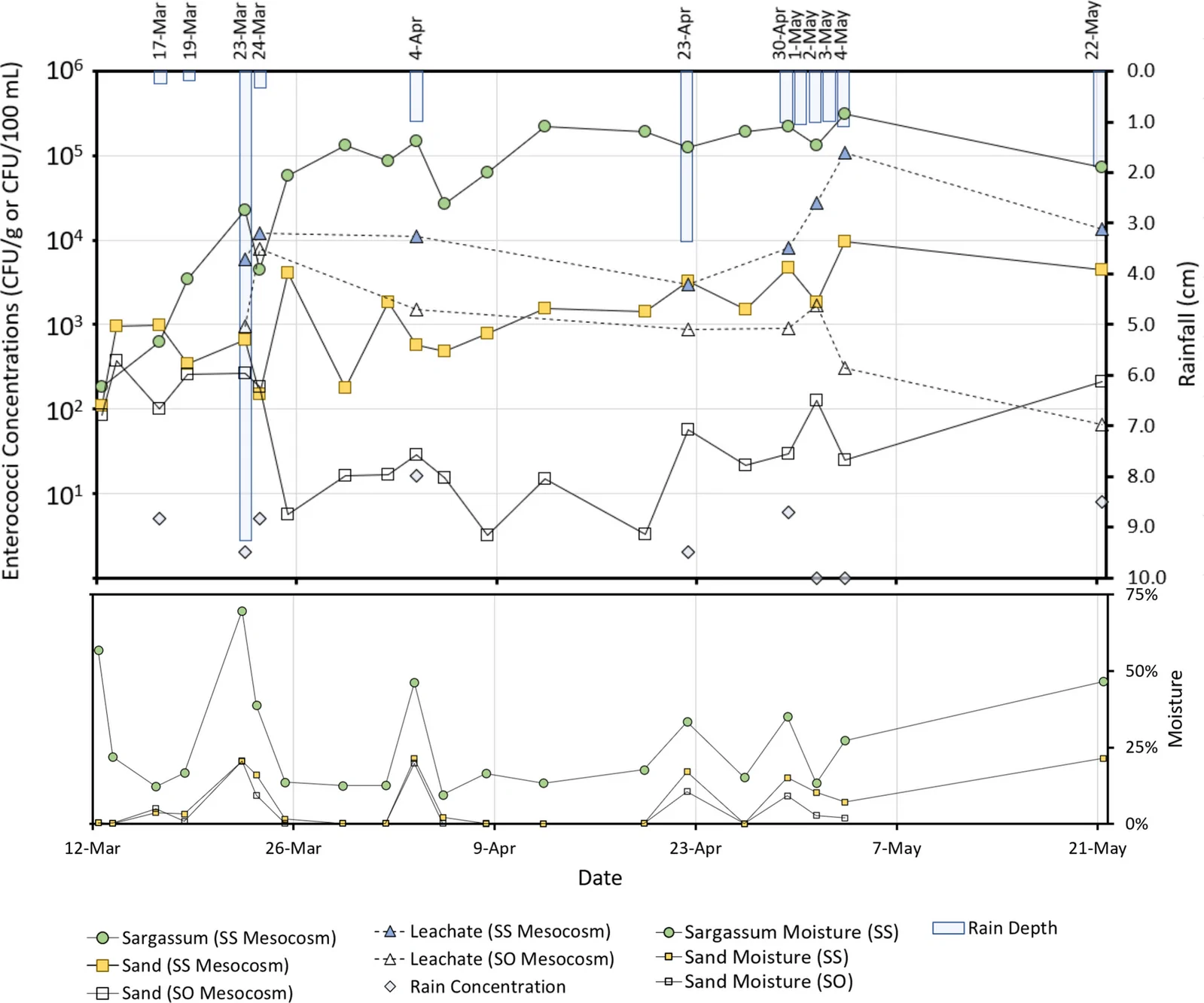
Time series plot of results from SS (symbols colored) and SO (symbols not colored) mesocosms. Units for sargassum and sand in CFU/g. Units for leachates and rain in CFU/100 mL. Rainfall depth in units of cm. Moisture content in units of percent

The relative concentration of enterococci in sargassum versus SS sand and versus SO sand is reflected in the moisture content of the sand (Fig. 3). Among the three sample types, sargassum had the highest average moisture content (26.4%) relative to SS sand (9.5%, *p* < 0.001) and SO sand (5.5%, *p* < 0.001). The difference in moisture content between SS and SO sand was not statistically significant (*p* = 0.22). Plotting results in time series (Fig. 4) show a significant increase in enterococci concentrations in the sargassum from an initial concentration at about 10^2^ increasing over three orders of magnitude to values above 10^5^ CFU/g. This increase in enterococci within the sargassum appears to respond to the moisture content. Each spike in moisture content is followed by a rise in enterococci concentrations up to a plateau. The increase in the enterococci is also reflected in the sand enterococci levels. In the SS mesocosms, the enterococci concentrations in the sand increased two orders of magnitude from 10^2^ to 10^4^ CFU/g. The increase in the sand generally followed the increase in the sargassum but with levels about an order of magnitude lower in the sand relative to the sargassum. The sand in the SO mesocosms showed lower levels of enterococci dropping from 10^2^ to 10^1^ CFU/g and then rebounding back to 10^2^ CFU/g towards the latter half of the study during times of moderate rainfall quantities. The significant difference between enterococci levels in sand between the SS and SO mesocosms (by two orders of magnitude) provides evidence that sargassum contributes towards higher enterococci levels in sand.

Similarly for the water phase, the leachate from the SS mesocosm was generally about one order of magnitude higher than the leachate from the SO mesocosm. Again, results suggest that the presence of sargassum increases the levels of enterococci in the leachate. The only exception was for a small rainfall event on March 24 for which the leachate from the SS mesocosm was only about 50% higher than the leachate from the SO mesocosm. The March 24 event followed a very large rainfall event on March 23 which suggests that the enterococci may have been flushed out from the sargassum as observed from the drop of enterococci in sargassum on March 24.

The enterococci in the leachates are contrasted by the enterococci measured in the rainfall, which was consistently low, although measurable. We attribute the measurable levels of enterococci in the rainwater to non-sterile conditions of rainfall and the possible influence of insects, as we would occasionally find dead mosquitoes within the rain gauge. Regardless, the levels of enterococci in rainfall are considered negligible, usually two orders of magnitude lower than the levels observed in the SO leachate. The rainfall therefore does not directly contribute significantly to the enterococci in the system. However, the moisture from the rainfall, appears to indirectly contribute through regrowth of enterococci in the sargassum and in the sand.

**Fig. 5:**
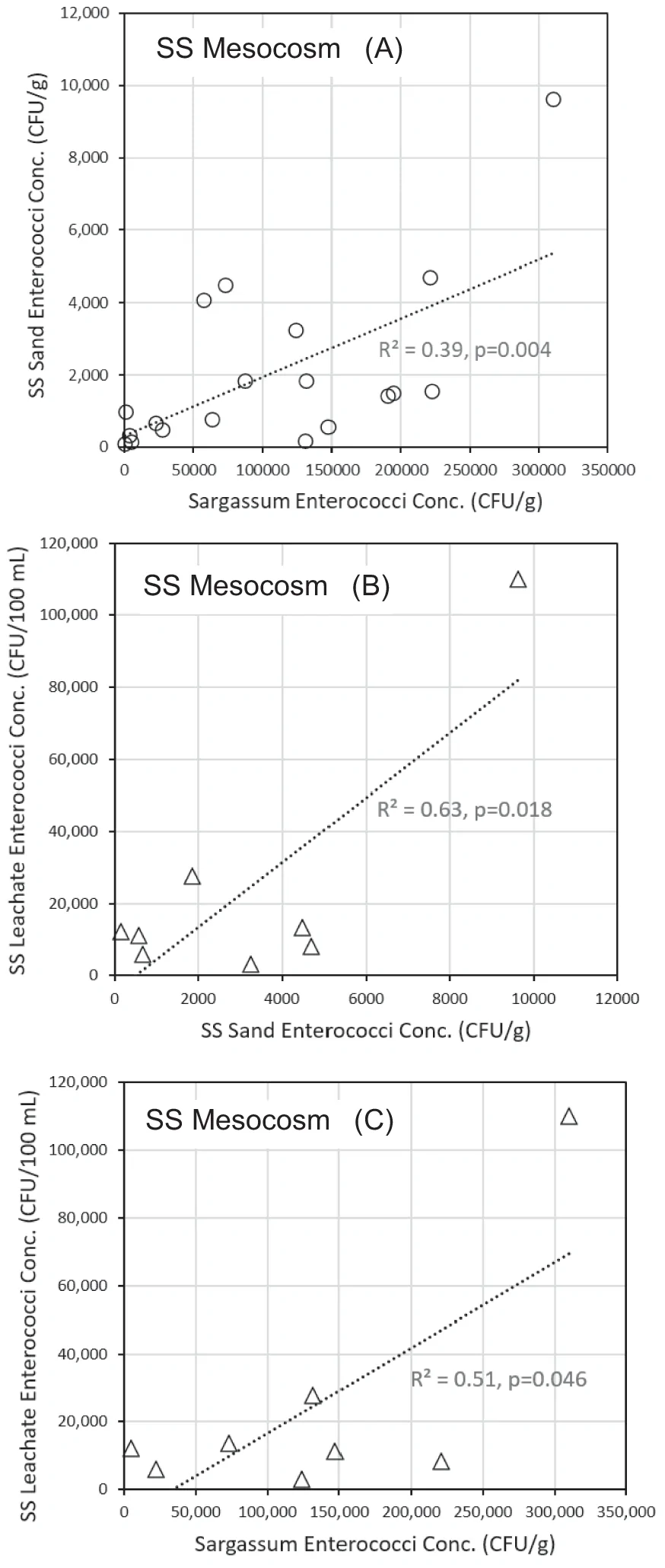
Correlations of enterococci levels within the SS mesocosms (**A**) between sargassum and sand, (**B**) between sand and leachate, and (**C**) between sargassum and leachate

Pearson correlations were significant only for the SS mesocosms (Table 1) when comparing the enterococci levels between the different matrices (Fig. 5A, B, and C). For the SS mesocosms the correlations were moderate and significant between sargassum and SS sand (*R*^2^ = 0.39, *p* = 0.004), between SS sand and SS leachate (*R*^2^ = 0.63, *p* = 0.018), and between sargassum and SS leachate (*R*^2^ = 0.51, *p* = 0.046). In contrast, for the SO mesocosms the correlations were weak and not significant (*R*^2^ = 0.05, *p* = 0.577 for SO sand and SO leachate). When making comparisons across mesocosms, no significant correlations were observed between SS and SO sand (*R*^2^ = 0.04, *p* = 0.387) and between SS leachate and SO leachate (*R*^2^ = 0.04, *p* = 0.623).

**Table 1:**
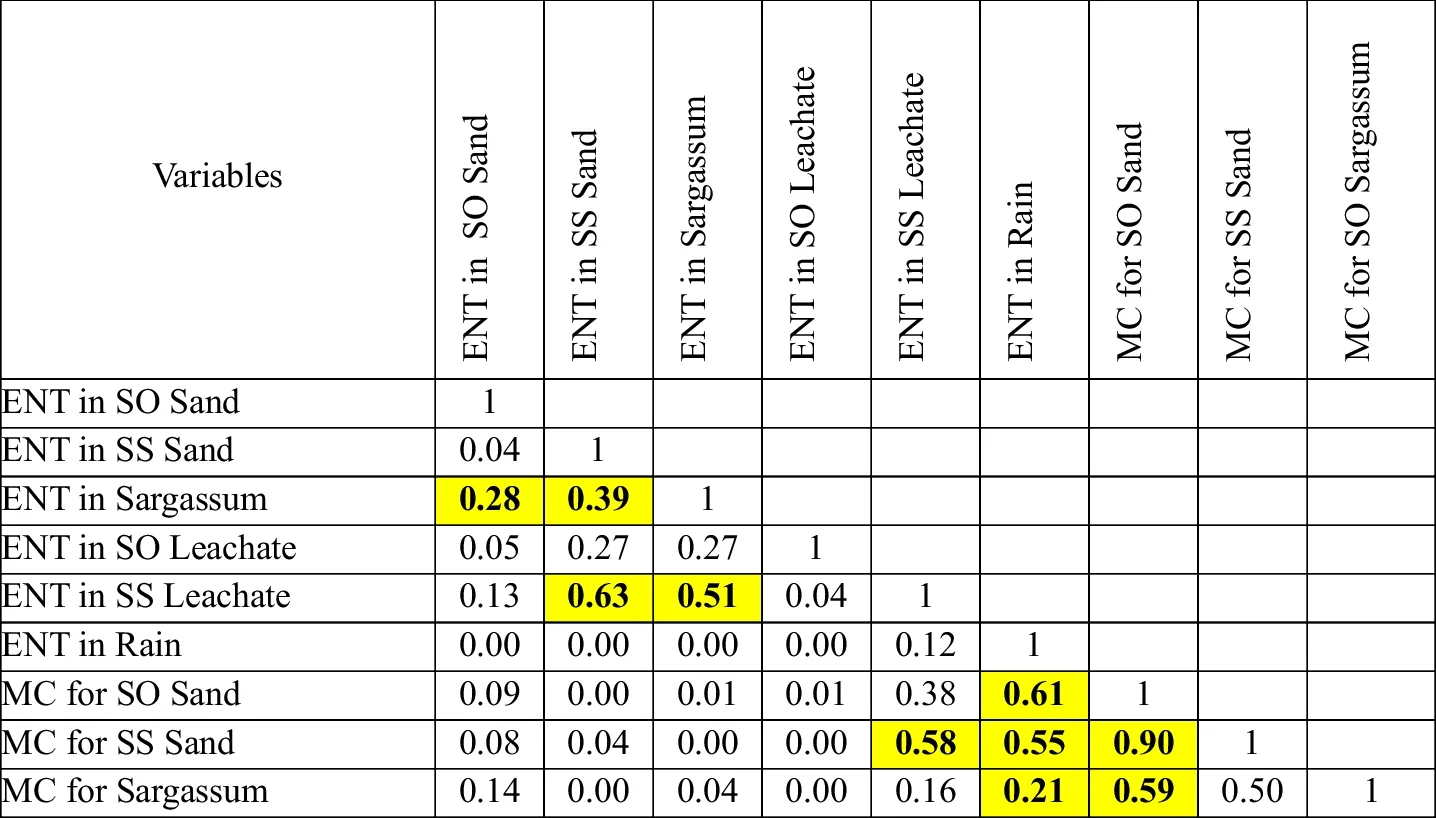
Pearson correlation matrix (*R*^2^) among enterococci concentrations (ENT) and moisture content (MC). Correlations with a yellow background and bold font are statistically significant at *p* < 0.05

Overall, the enterococci levels in the SS mesocosms were correlated among the different matrices (sargassum, sand, leachate), likely influenced by the extremely high levels observed in the sargassum.

## Discussion

### Support for enterococci growth in sargassum and sand

Results show that enterococci persist and grow in sargassum. During rain events, the enterococci may transfer from sargassum into the liquid leachates that infiltrate through the sand, or the enterococci may transfer from the sargassum to sand and then to the leachate. Through either route, the excess enterococci observed in the sand in the SS mesocosm (compared to the SO mesocosm) most likely comes from the sargassum through the action of rainwater infiltration. There is also evidence for the growth of enterococci in the sand. Increases were observed in the sand enterococci concentration in the SS mesocosms between rainfall events. These increases generally followed the increases in the enterococci levels in sargassum, suggesting that enterococci were multiplying in both the sargassum and sand media between rainfall events, since there was no infiltration to facilitate the transfer of enterococci from the sargassum to the sand below.

There are several potential reasons for the growth of enterococci in sargassum and sand. First, the sargassum layer can provide protection from solar radiation (Beckinghausen et al., 2014). Sassoubre et al. (2012) documented photoinactivation of enterococci through the action of sunlight. The sargassum layer overtopping the sand layer provides shielding from the impacts of sunlight, protecting enterococci in lower layers at the beach. Second, the sargassum itself can potentially serve as a substrate for enterococci growth. Studies have shown that wrack, and in particular brown macroalgae and seagrasses (Kalvaitienė et al., 2023), serve as environments for biofilm growth and provide protection against predation (Egan et al., 2013). As sargassum decays it can release nitrogen and phosphorus making it available for utilization by enterococci within biofilms. Moreover, these nutrients can be flushed into the sand below providing for a transfer of nutrients that will be available for enterococci residing in sand, where enterococci are also known to reside in biofilms (Piggot et al., 2012). Third, sargassum stabilizes the fluctuating ambient conditions found in sand by buffering swings in temperature and moisture content (Maurer et al., 2022). This stabilization helps to maintain temperatures within the wide growth range of enterococci (10 to 45 °C) (Ramsey et al., 2014). Similarly, the stabilization of moisture content also facilitates the long-term survival of enterococci beyond their innate high tolerance to desiccation (Kramer et al., 2006). It is possible that the wetting and drying cycles associated with the intermittent rain are conducive to enterococci regrowth. During the drying cycle competing bacteria may not survive creating an optimal environment where enterococci are one of the few survivors, facilitating regrowth when moisture is again restored. The influence of moisture restoration on increasing FIB levels in beach sands has been shown through field studies that have found higher levels of FIB in beach sands with higher moisture (Yamahara et al., 2012). Research is needed to evaluate the dynamics of the complex microbiome of sargassum (Cox et al., 2025) in efforts to evaluate competition among microbial communities and conditions that allow for the growth of enterococci.

### Support for sargassum to contribute enterococci in leachates

The enterococci observed in the leachates from the SS mesocosm was moderately and significantly correlated with enterococci levels in the sargassum and with enterococci levels in the sand. It is likely that during rainfall events, infiltration carries enterococci from the sargassum into the sand and ultimately into the leachate. However, visual inspection of the plots identified one potentially influential observation. Sensitivity analysis showed that the correlations were no longer statistically significant after excluding this maximum value, suggesting that the association should be interpreted with caution.

Since leachates were generated only during rainfall events and were discarded after each day, we are unable to determine whether the water would be able to support the growth of enterococci. Future research should keep the leachates within the reservoir over time (without discarding) to determine whether levels of enterococci increase or decrease over time between storm events. A study conducted in tropical waters of Malaysia showed that *Enterococcus faecium* grew in seawater, and this growth was promoted by warming water from 25 to 37 °C (Wong et al., 2022). In contrast other studies, which seeded enterococci into treated wastewater documented temperature dependent decay, but in this case, decay was more rapid at the warmer temperature (25 °C) compared to the cooler temperature (4 °C) (Chern et al., 2022). Of interest would be to determine whether there are differences in decay or growth of enterococci in water leachates from SO versus the SS mesocosms.

### Results in the context of beach management

Results suggest that enterococci multiply in sargassum resulting in extremely high levels on the order of 5-log_10_. These extremely high levels in the sargassum do impact the sand and ultimately the leachate produced during rainfall events and during tidal washing. For beaches that suffer from elevated levels of enterococci in water or sand, results suggest that managing the sargassum, a major reservoir of enterococci, can reduce enterococci levels. It is common in some areas to integrate sargassum into beach sand (Abdool-Ghany et al., 2023). Integration is the process of cutting the sargassum and mixing it with the underlying sand. Field studies of beaches that implement this practice have been observed to have high frequencies of beach closures depending upon waves and the timing of the tides which influence the washing dynamics of sargassum that strands along beaches (Abdool-Ghany et al., 2022). Because the integration of sargassum into sand is associated with high levels of enterococci, such practices of sargassum integration should be discouraged. When sargassum strandings are excessive, the sargassum should be removed (not integrated) and handled offsite.

### Recommendations

The mesocosms used in the current study simulated sargassum strandings that were not impacted by tidal washing. At beaches, sargassum will undergo considerable tidal washing. It would be interesting to evaluate enterococci levels during simulated tidal washing scenarios coupled with natural rainfall and sunlight. Collected water leachates should be evaluated to determine whether the enterococci transferred to the water decays or grows.

In efforts to assess microbial dynamics, culture methods should be implemented to document the enterococci species, as survival, persistence, and regrowth are likely influenced by the dominating enterococci species. In addition, results should be augmented with studies that also document the microbial community as changes have been observed in the microbial community as the sargassum decays (Abdool-Ghany et al., 2025). We also recommend measurements of nutrient releases and evaluating the effect of these nutrient releases on sargassum enterococci levels. A model should be developed that integrates information known about the effects of sunlight, temperature, moisture content, and nutrients, coupled with simulations of rainfall events and wave action. Such a model would be useful to confirm the mechanistic understanding of the processes underlying the changes in enterococci levels.

### Limitations

The mesocosm experiments were conducted outdoors and thus the conditions were not sterile and could have been influenced by microbial conditions of the outdoor environment. This was observed directly in the current study as the rainwater quality was likely influenced by insects. In addition, sensitivity analysis indicated that the significant correlations observed were driven by maximum values of enterococci concentrations. Experiments should be repeated to confirm the observed correlations.

## Conclusions

Results from this study provide evidence that enterococci persist and multiply in sargassum. The enterococci in the sargassum contribute to elevated levels of enterococci in beach sands and in rainwater leachates. Beach sand without sargassum also showed evidence of enterococci persistence and regrowth, but not to the same extent as sands impacted by sargassum. The results of this study support the hypothesis that sargassum acts as both a source and amplifier of enterococci, and that its presence creates conditions favorable for bacterial persistence. More research is needed to understand the role of moisture and nutrients that maintain a microbial community conducive to the proliferation of enterococci. Overall, results from this study are of significance to beach managers who aim to keep beaches open for recreational swimming and sand contact activities. Understanding that sargassum is a reservoir and incubator for enterococci will facilitate management decisions to reduce excessive levels of enterococci in the recreational environment.

## Supplementary Information

Below is the link to the electronic supplementary material.

ESM 1 DOCX (31.0 KB)

## Data Availability

The data analyzed in the current study are available from the corresponding author on reasonable request.
