# Supplementary material for: Proliferation of enterococci in sargassum can contribute towards sand and water quality impairment
Source: Environ Monit Assess. 2026 Sep 2;198(9):1025. doi: 10.1007/s10661-026-15872-3 (PMC13538262; doi:10.1007/s10661-026-15872-3)
Supplement: Supplementary file 1 [file 10661_2026_15872_MOESM1_ESM.docx]

**Supplement for**

Proliferation of Enterococci in Sargassum

Can Contribute Towards Sand and Water Quality Impairment

Authors: Afeefa A. Abdool-Ghany^1^, Kevin Huang^1^, Matthew Roca^1^, Eric Saba^1^,

Jiayu Li^3^, Helena M. Solo-Gabriele^1^

^1^Department of Chemical, Environmental, and Materials Engineering, University of Miami, Coral Gables, Florida, USA

^2^Department of Chemistry and Biochemistry, Florida International University, Miami, Florida, USA

^3^Department of Mechanical and Aerospace Engineering, University of Miami, Coral Gables, Florida, USA

For consideration in:

*Environmental Monitoring and Assessment*

Version Date: August 1, 2026

Table S-1. Rainfall data during the mesocosm experiment. Days highlighted in yellow correspond to days when samples were collected.

| DAY | Date | Time of Readings | Rain gauge 1 (in) | Rain gauge 2 (in) | Average Rainfall (in) |
| --- | --- | --- | --- | --- | --- |
| 0 | 3/13/2024 | 4:45:00 PM | 0 | 0 | 0 |
| 1 | 3/14/2024 | 9:50:00 AM | 0 | 0 | 0 |
| 2 | 3/15/2024 | 9:10:00 AM | 0 | 0 | 0 |
| 3 | 3/16/2024 | 8:50:00 AM | 0 | 0 | 0 |
| 4 | 3/17/2024 | 9:55:00 AM | 0.09 | 0.10 | 0.095 |
| 5 | 3/18/2024 | 9:12:00 AM | 0 | 0 | 0 |
| 6 | 3/19/2024 | 9:22:00 AM | 0.07 | 0.08 | 0.075 |
| 7 | 3/20/2024 | 9:30:00 AM | 0 | 0 | 0 |
| 8 | 3/21/2024 | 9:50:00 AM | 0 | 0 | 0 |
| 9 | 3/22/2024 | 9:40:00 AM | 0 | 0 | 0 |
| 10 | 3/23/2024 | 9:58:00 AM | 3.69 | 3.61 | 3.65 |
| 11 | 3/24/2024 | 9:03:00 AM | 0.13 | 0.12 | 0.125 |
| 12 | 3/25/2024 | 9:00:00 AM | 0 | 0 | 0 |
| 13 | 3/26/2024 | 9:15:00 AM | 0 | 0 | 0 |
| 14 | 3/27/2024 | 9:55:00 AM | 0 | 0 | 0 |
| 15 | 3/28/2024 | 9:19:00 AM | 0 | 0 | 0 |
| 16 | 3/29/2024 | 10:01:00 AM | 0 | 0 | 0 |
| 17 | 3/30/2024 | 9:00:00 AM | 0 | 0 | 0 |
| 18 | 3/31/2024 | 10:00:00 AM | 0 | 0 | 0 |
| 19 | 4/1/2024 | 9:50:00 AM | 0 | 0 | 0 |
| 20 | 4/2/2024 | 9:15:00 AM | 0 | 0 | 0 |
| 21 | 4/3/2024 | 9:51:00 AM | 0 | 0 | 0 |
| 22 | 4/4/2024 | 9:30:00 AM | 0.39 | 0.385 | 0.3875 |
| 23 | 4/5/2024 | 9:56:00 AM | 0 | 0 | 0 |
| 24 | 4/6/2024 | 9:55:00 AM | 0 | 0 | 0 |

Table S-1. (continued)

| DAY | Date | Time of Readings | Rain gauge 1 (in) | Rain gauge 2 (in) | Average Rainfall (in) |
| --- | --- | --- | --- | --- | --- |
| 25 | 4/7/2024 | 10:00:00 AM | 0 | 0 | 0 |
| 26 | 4/8/2024 | 9:45:00 AM | 0 | 0 | 0 |
| 27 | 4/9/2024 | 8:52:00 AM | 0 | 0 | 0 |
| 28 | 4/10/2024 | 9:50:00 AM | 0 | 0 | 0 |
| 29 | 4/11/2024 | 9:50:00 AM | 0 | 0 | 0 |
| 30 | 4/12/2024 | 9:55:00 AM | 0 | 0 | 0 |
| 31 | 4/13/2024 | 9:08:00 AM | 0 | 0 | 0 |
| 32 | 4/14/2024 | 10:00:00 AM | 0 | 0 | 0 |
| 33 | 4/15/2024 | 9:58:00 AM | 0 | 0 | 0 |
| 34 | 4/16/2024 | 10:00:00 AM | 0 | 0 | 0 |
| 35 | 4/17/2024 | 10:00:00 AM | 0 | 0 | 0 |
| 36 | 4/18/2024 | 9:50:00 AM | 0 | 0 | 0 |
| 37 | 4/19/2024 | 9:58:00 AM | 0 | 0 | 0 |
| 38 | 4/20/2024 | 9:18:00 AM | 0 | 0 | 0 |
| 39 | 4/21/2024 | 10:00:00 AM | 0 | 0 | 0 |
| 40 | 4/22/2024 | 10:00:00 AM | 0 | 0 | 0 |
| 41 | 4/23/2024 | 9:45:00 AM | 1.31 | 1.34 | 1.325 |
| 42 | 4/24/2024 | 10:00:00 AM | 0 | 0 | 0 |
| 43 | 4/25/2024 | 10:00:00 AM | 0 | 0 | 0 |
| 44 | 4/26/2024 | 9:55:00 AM | 0 | 0 | 0 |
| 45 | 4/27/2024 | 9:40:00 AM | 0 | 0 | 0 |
| 46 | 4/28/2024 | 9:45:00 AM | 0 | 0 | 0 |
| 47 | 4/29/2024 | 10:00:00 AM | 0 | 0 | 0 |
| 48 | 4/30/2024 | 11:20:00 AM | 0.40 | 0.395 | 0.3975 |
| 49 | 5/1/2024 | 8:10:00 AM | 0.41 | 0.42 | 0.415 |

Table S-1. (continued)

| DAY | Date | Time of Readings | Rain gauge 1 (in) | Rain gauge 2 (in) | Average Rainfall (in) |
| --- | --- | --- | --- | --- | --- |
| 50 | 5/2/2024 | 9:45:00 AM | 0.40 | 0.40 | 0.40 |
| 51 | 5/3/2024 | 10:15:00 AM | 0.4 | 0.38 | 0.39 |
| 52 | 5/4/2024 | 9:15:00 AM | 0.43 | 0.425 | 0.4275 |
| 53 | 5/5/2024 | 9:45:00 AM | 0 | 0 | 0 |
| 54 | 5/6/2024 | 10:30:00 AM | 0 | 0 | 0 |
| 55 | 5/7/2024 | 10:15:00 AM | 0 | 0 | 0 |
| 56 | 5/8/2024 | 10:00:00 AM | 0 | 0 | 0 |
| 57 | 5/9/2024 | 9:55:00 AM | 0 | 0 | 0 |
| 58 | 5/10/2024 | 10:00:00 AM | 0 | 0 | 0 |
| 59 | 5/11/2024 | 9:07:00 AM | 0 | 0 | 0 |
| 60 | 5/12/2024 | 10:00:00 AM | 0 | 0 | 0 |
| 61 | 5/13/2024 | 10:15:00 AM | 0 | 0 | 0 |
| 62 | 5/14/2024 | 9:20:00 AM | 0 | 0 | 0 |
| 63 | 5/15/2024 | 9:00:00 AM | 0 | 0 | 0 |
| 64 | 5/16/2024 | 10:00:00 AM | 0 | 0 | 0 |
| 65 | 5/17/2024 | 6:27:00 AM | 0 | 0 | 0 |
| 66 | 5/18/2024 | 10:40:00 AM | 0 | 0 | 0 |
| 67 | 5/19/2024 | 11:10:00 AM | 0 | 0 | 0 |
| 68 | 5/20/2024 | 10:00:00 AM | 0 | 0 | 0 |
| 69 | 5/21/2024 | 6:30:00 AM | 0 | 0 | 0 |
| 70 | 5/22/2024 | 8:30:00 AM | 0.77 | 0.71 | 0.74 |

Table S-2. Raw enterococci (ENT) and moisture content (MC) data collected during mesocosm experiments. SO and SS refer to “sand only” and “sargassum + sand” mesocosms.

| Date  M/DD/YYYY | Enterococci Concentrations | | | | | | Moisture Content | | |
| --- | --- | --- | --- | --- | --- | --- | --- | --- | --- |
|  | Rain ENT (CFU/100 mL) | SO Sand ENT (CFU/dry g) | SS Sand ENT (CFU/dry g) | Sargassum ENT  (CFU/dry g) | SO Leachate ENT (CFU/100mL) | SS Leachate ENT (CFU/100mL) | SO MC of Sand | SS MC of Sand | SS MC of Sargassum |
| 3/13/2024 | 0 | 84.3 | 109.7 | 180.0 |  |  | 0.4% | 0.5% | 56.8% |
| 3/16/2024 | 0 | 373.6 | 948.9 |  |  |  | 0.3% | 0.2% | 21.9% |
| 3/17/2024 | 5 | 100.3 | 984.1 | 621.7 |  |  | 5.0% | 3.7% | 12.2% |
| 3/19/2024 | 0 | 253.7 | 343.9 | 3,399 |  |  | 0.8% | 3.2% | 16.7% |
| 3/23/2024 | 2 | 263.1 | 665.8 | 22,470 | 952 | 6,000 | 20.5% | 20.6% | 69.7% |
| 3/24/2024 | 5 | 179.9 | 149.4 | 4,429 | 7,920 | 12,080 | 9.3% | 16.0% | 38.8% |
| 3/26/2024 | 0 | 5.5 | 4085 | 57,380 |  |  | 0.2% | 1.6% | 13.7% |
| 3/30/2024 | 0 | 15.9 | 178.8 | 131,100 |  |  | 0.2% | 0.1% | 12.5% |
| 4/2/2024 | 0 | 16.7 | 1,852 | 87,420 |  |  | 0.2% | 0.2% | 12.6% |
| 4/4/2024 | 16 | 28.3 | 573.7 | 147,000 | 1,507 | 11,228 | 19.8% | 21.4% | 46.2% |
| 4/6/2024 | 0 | 15.1 | 487.8 | 26,958 |  |  | 0.1% | 2.1% | 9.5% |
| 4/9/2024 | 0 | 3.2 | 778.8 | 63,460 |  |  | 0.1% | 0.1% | 16.5% |
| 4/13/2024 | 0 | 14.7 | 1,549 | 222,145 |  |  | 0.1% | 0.1% | 13.4% |
| 4/20/2024 | 0 | 3.3 | 1,425 | 190,056 |  |  | 0.2% | 0.2% | 17.7% |
| 4/23/2024 | 2 | 56.7 | 3,264 | 124,159 | 882 | 3,015 | 10.6% | 17.1% | 33.4% |
| 4/27/2024 | 0 | 21.2 | 1,511 | 194,637 |  |  | 0.1% | 0.1% | 15.2% |
| 4/30/2024 | 6 | 29.0 | 4,697 | 220,629 | 893 | 8,179 | 9.2% | 15.1% | 35.1% |
| 5/2/2024 | 1 | 125.2 | 1,849 | 131262 | 1,704 | 27,638 | 2.8% | 10.3% | 13.3% |
| 5/4/2024 | 1 | 24.8 | 9,634 | 310104 | 306 | 110,167 | 1.9% | 7.2% | 27.3% |
| 5/22/2024 | 8 | 212.7 | 4,496 | 72915 | 65 | 13,447 | 17.3% | 21.5% | 46.5% |
